# Developing an Embedding, Koopman and Autoencoder Technologies-Based Multi-Omics Time Series Predictive Model (EKATP) for Systems Biology research

**DOI:** 10.3389/fgene.2021.761629

**Published:** 2021-10-26

**Authors:** Suran Liu, Yujie You, Zhaoqi Tong, Le Zhang

**Affiliations:** ^1^ College of Computer Science, Sichuan University, Chengdu, China; ^2^ College of Software Engineering, Sichuan University, Chengdu, China

**Keywords:** multi-omics, time series, embedding, Koopman, deep learning

## Abstract

It is very important for systems biologists to predict the state of the multi-omics time series for disease occurrence and health detection. However, it is difficult to make the prediction due to the high-dimensional, nonlinear and noisy characteristics of the multi-omics time series data. For this reason, this study innovatively proposes an Embedding, Koopman and Autoencoder technologies-based multi-omics time series predictive model (EKATP) to predict the future state of a high-dimensional nonlinear multi-omics time series. We evaluate this EKATP by using a genomics time series with chaotic behavior, a proteomics time series with oscillating behavior and a metabolomics time series with flow behavior. The computational experiments demonstrate that our proposed EKATP can substantially improve the accuracy, robustness and generalizability to predict the future state of a time series for multi-omics data.

## Introduction

Currently, the prediction of multi-omics time series states is one of the trending areas in systems biology research (Zhang et al., 2019a). In particular, the development of high-throughput technology (Soon et al., 2013) has produced a large-scale time series multi-omics state (Liang and Kelemen 2017a), including genomics (Lockhart and Winzeler 2000), proteomics (Tyers and Mann, 2003), metabolomics (Weckwerth 2003) and more. Previous studies usually employed differential equation (Eisenhammer et al., 1991; Zhang et al., 2016; Zhang and Zhang 2017; Liu G.-D. et al., 2020) based models to abstract and formalise multi-omics time series data (Bianconi et al., 2020). Then, it became possible to explore the time-varying connections and predict their future state (Ji et al., 2017) by solving these differential equations. In particular, predicting multi-omics time series states can not only discover dynamic information for biological entities, such as genes, proteins and metabolites, but also explore complicated biological interactions and the pathogenesis of diseases (Liang and Kelemen, 2017b).

However, a multi-omics time series usually has high dimensions (Perez-Riverol et al., 2017), complicated interaction relationships (Fischer 2008) and inevitable noise (Fischer 2008; Tsimring 2014). Thus, when we employ differential equations to model the multi-omics time series state, it is hard for us to solve these equations due to their high dimensionality and nonlinear characteristics (Bianconi et al., 2020). For these reasons, the way to predict the future state of a multi-omics time series by solving these complicated nonlinear differential equations has already become challenging work.

Recently, future state prediction for a multi-omics time series has been widely studied by computational biologists. For genomic studies, we usually use a gene expression time series to develop gene regulatory networks (Davidson and Levin 2005; Zhang et al., 2018; Xiao et al., 2020; Zhang et al., 2020; Xiao et al., 2021; Zhang et al., 2021a). However, since the gene regulatory network is a complex high-dimensional nonlinear system (Zhang et al., 2012a), it often produces chaotic phenomena (Levnajić and Tadić 2010), which not only play an important role in maintaining stable gene expression patterns (Sevim and Rikvold 2008) but also are closely related to the occurrence of diseases (Suzuki et al., 2016). Usually, we employ the Lorentz system (Lorenz 1963) to describe the chaotic phenomenon. However, it is inaccurate to predict the future state of genomics time series with nonlinear complicated interactions because the Lorentz system is not good at processing nonlinear complicated interactions (Lai et al., 2018). Currently, delay embedding theory (Sauer et al., 1991; Holmes et al., 2012) is commonly used to transform the spatial information (complicated interactions) into temporal information (the future state of the time series (Chen et al., 2020)) for dimensional reduction (Gao et al., 2017; Li et al., 2017; Xia et al., 2017; Zhang et al., 2019b; Zhang et al., 2019c; Wu et al., 2020; You et al., 2020; Zhang et al., 2021b), whereas Koopman theory (Koopman, 1931) can switch the nonlinear system into a linear system to reduce computing cost. Therefore, our first research question asks if we can develop such a time series predictive model that integrates the Lorentz system with delay embedding and Koopman theory to accurately predict the future state of genomics time series with chaotic behavior.

For proteomics studies, we usually use proteomic time series data to infer protein–protein interactions (PPIs) (Wu et al., 2009). Currently, we employ mass spectrometry technology (Mann et al., 2001) to obtain proteomics time series data. However, since it is unstable to have time-course experimental data by mass spectrometry technology, proteomics time series data are prone to oscillating behavior (Iuchi et al., 2018). Previously, we employed a nonlinear pendulum system (Hirsch 1974) to describe the oscillation behavior, though it was subjected to overfitting under a strong noise environment. Since the conjugate form of delay embedding (Sauer et al., 1991; Holmes et al., 2012) can ensure the reversibility of the time series predictive model (Chen et al., 2020) and reduce the impact of noise on prediction to a certain extent, our second research question asks if we can develop such a time series predictive model that can integrate a nonlinear pendulum system with delay embedding to accurately predict the state of proteomics time series with oscillating behavior.

For metabolomics studies, we usually use metabolic time series data that represent the flow behavior of biological fluids (serum, cerebrospinal fluid, etc.) to discover key metabolites in biological fluids (Zhang et al., 2012b). A previous study (Noack et al., 2003) always employed a nonlinear biological fluid system to describe metabolic time series data. However, because most nonlinear fluid flow systems have high dimensions (Lusch et al., 2018), we not only have difficulty selecting features from high-dimensional metabolic time series data but also impede progress because of time-consuming computing (Wang et al., 2021). Currently, since neural networks (Wang et al., 2014) can decrease the computing cost (Song et al., 2017) by dimensional reduction for time series data (Hinton and Salakhutdinov, 2006), our third research question asks if we can develop such a time series predictive model that integrates a nonlinear fluid flow system with a neural network to predict the future state of the metabolomics time series accurately and quickly with flow behaviour.

To answer the above three research questions, this study innovatively develops an Embedding, Koopman and Autoencoder technologies-based multi-omics time series predictive model (EKATP) to predict the future state of the time series for the corresponding genomics, proteomics and metabolomics datasets. Compared with previous approaches (Lusch et al., 2018; Azencot et al., 2020), the contributions of the study are summarised as follows. First, we select key features from a high-dimensional nonlinear state by integrating a neural network with the delay embedding theory. Second, we switch the nonlinear system with a linear system to reduce the computing cost by the Koopman theory. Finally, we develop a neural network and delay embedding theory-based model for reversible mapping between a high-dimensional nonlinear system and a low-dimensional linear system, thereby improving the accuracy and robustness of prediction.

The rest of the manuscript is organised as follows. *Related Works* mainly describes the related work for Autoencoder, delay embedding theory and Koopman theory. *Materials and Methods* introduces the architecture of the EKATP and the related procedure. *Experiments* describes the computational experiments. Finally, we conclude the study and discuss the future work.

## Related Works

Supplementary Presentation S1 details the related theory and existing research of the Autoencoder, delay embedding theory and Koopman theory.

## Materials and Methods

**FIGURE 1:**
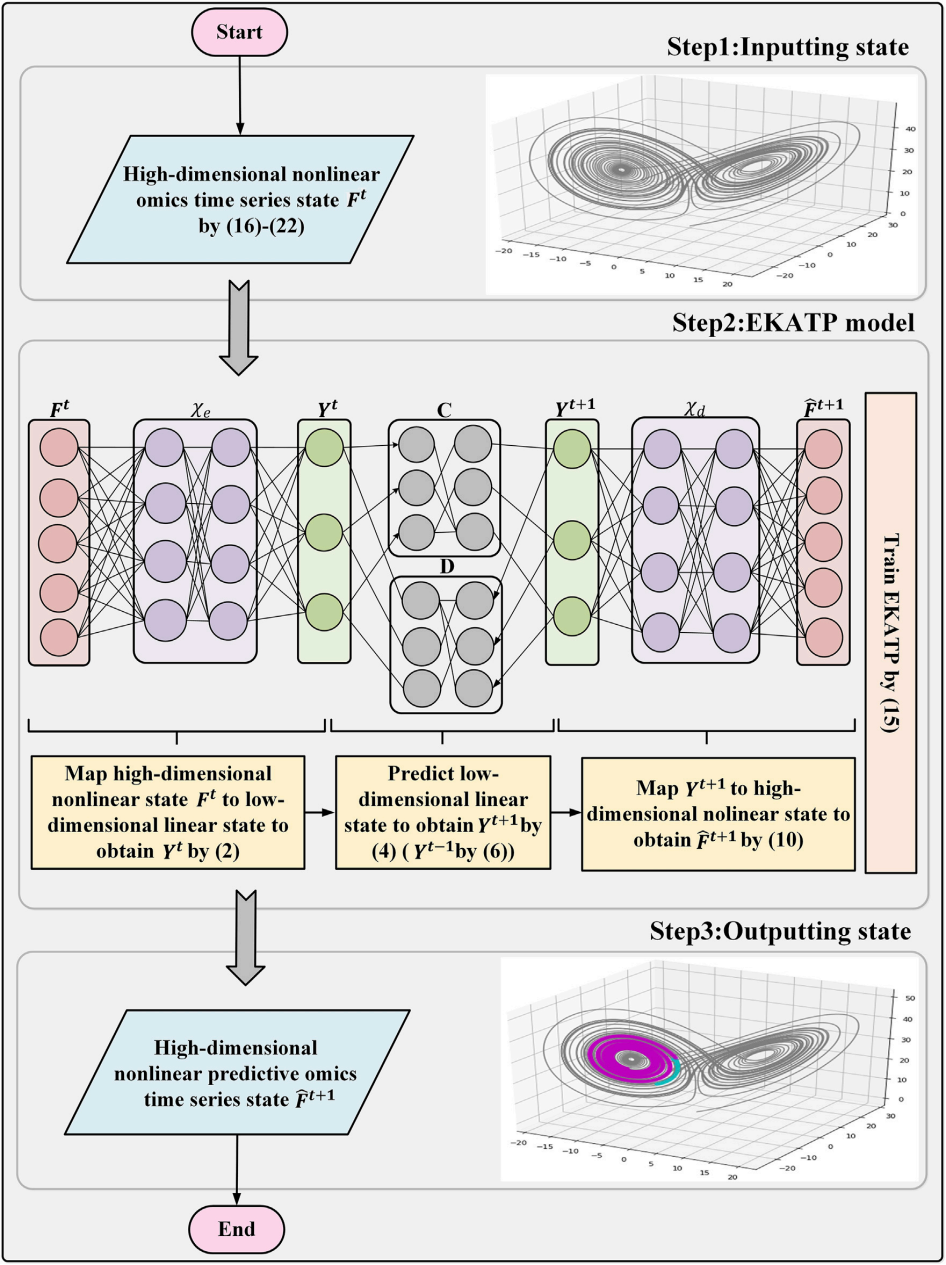
EKATP workflow.

Figure 1 describes the workflow of the EKATP.

### Problem and Definitions

Given a set of high-dimensional nonlinear multi-omics time series states $F=\left(F^{1},F^{2},\ldots ,F^{T}\right)$ , where T represents the total step, the time series state at t can be described as $F^{t}={\left(f_{1}^{t},f_{2}^{t},\ldots f_{n}^{t}\right)}^{\prime }$ , where n represents the dimension of the time series state, “ ' ”, as the transpose of a vector. Our goal is to predict the future state of the multi-omics time series. Next, we detail how to develop an EKATP as follows.

### Autoencoding Observations

Since an EKATP is based on the Autoencoder framework, we employ Eq. 1 to define the objective function for Autoencoder ( $L_{id}$ ).

$$L_{id}={\left\|{\hat{F}}^{t}-F^{t}\right\|}_{\text{MSE}}$$ (1)

Here, ${\hat{F}}^{t}$ is the reconstructed high-dimensional time series state according to encoder ( $\chi_{e}$ ) and decoder ( $\chi_{d}$ ) of Autoencoder (Supplementary Presentation S1). ${\left|\left|\cdot \right|\right|}_{\text{MSE}}$ denotes the mean squared error (MSE), which presents the expected value of the square of the difference between the predicted value and the true value. This loss function term enables us to construct an Autoencoder model that satisfies $\chi_{d}\circ \chi_{e}\approx id$ , the identity.

### Delay Embedding

According to the description in the delay embedding theory (Supplementary Presentation S1), we employ $\chi_{e}$ of the Autoencoder to approximate the delay embedding Φ , mapping the high-dimensional nonlinear input time series state $F^{t}$ back to the low-dimensional time series state $Y^{t}$ by Eq. 2,

$$Y^{t}={\left(y^{t},y^{t+1},...,y^{t+L-1}\right)}^{\prime }=\chi_{e}\left(F^{t}\right).$$ (2)

where L represents the dimension of the low-dimensional time series state. Similarly, the inverse mapping $\chi_{d}$ of mapping $\chi_{e}$ is used to approximate the conjugate form of delay embedding Φ , mapping the low-dimensional time series state back to the high-dimensional time series state by Eq. 3.

$${\hat{F}}^{t}={\left({\hat{f}}_{1}^{t},{\hat{f}}_{2}^{t},\ldots {\hat{f}}_{n}^{t}\right)}^{\prime }=\chi_{d}\left(Y^{t}\right).$$ (3)

### Linearized Representation of the Koopman Operator

Based on the Koopman theory discussed by Supplementary Presentation S1, we construct the finite dimensional linear matrix C (and matrix D ) to compute the forward (and backward) low-dimensional time series state. Equation 4 shows how to realize the forward prediction for low-dimensional time series state $Y^{t}$ to obtain $Y^{t+1}$ .

$$Y^{t+1}=CY^{t}.$$ (4)

Equation 4 can be expanded by Eq. 5.

$$\left[\begin{array}{c} y^{t+1}\\ y^{t+2}\\ y^{t+3}\\ \ldots \\ y^{t+L-1}\\ y^{t+L} \end{array}\right]=\left[\begin{array}{ccccc} 0 & 1 & \ldots & 0 & 0\\ 0 & 0 & \ldots & 0 & 0\\ 0 & 0 & \ldots & 0 & 0\\ \ldots & \ldots & \ldots & \ldots & \ldots \\ 0 & 0 & \ldots & 0 & 1\\ a_{1} & a_{2} & \ldots & a_{L-1} & a_{L} \end{array}\right]\left[\begin{array}{c} y^{t}\\ y^{t+1}\\ y^{t+2}\\ \ldots \\ y^{t+L-2}\\ y^{t+L-1} \end{array}\right].$$ (5)

Here, $a_{i}$ is the estimated parameter that needs training, and $a_{1}\neq 0$ . Equation 6 shows how to realize the backward prediction for a low-dimensional time series state $Y^{t}$ to obtain $Y^{t-1}$ .

$$Y^{t-1}=DY^{t}.$$ (6)

Equation 6 can be expanded by Eq. 7.

$$\left[\begin{array}{c} y^{t-1}\\ y^{t}\\ y^{t+1}\\ \ldots \\ y^{t+L-3}\\ y^{t+L-2} \end{array}\right]=\left[\begin{array}{ccccc} b_{1} & b_{2} & \ldots & b_{L-1} & b_{L}\\ 1 & 0 & \ldots & 0 & 0\\ 0 & 1 & \ldots & 0 & 0\\ \ldots & \ldots & \ldots & \ldots & \ldots \\ 0 & 0 & \ldots & 0 & 0\\ 0 & 0 & \ldots & 1 & 0 \end{array}\right]\left[\begin{array}{c} y^{t}\\ y^{t+1}\\ y^{t+2}\\ \ldots \\ y^{t+L-2}\\ y^{t+L-1} \end{array}\right]\;.$$ (7)

Here, $b_{i}$ is the estimated parameter that needs training, and $b_{L}\neq 0$ . Our goal is to optimise the parameters of the linear matrix C (and D ) of Eqs 5, 7 by model training.

### Forward and Backward Prediction

We make the k -steps forward prediction by Eq. 8 and backward prediction by Eq. 9 for the state of the low-dimensional time series $Y^{t}$ . After that, $\chi_{d}$ is used to map the low-dimensional predictive time series state back to the high-dimensional predictive time series state by Eq. 10,

$$Y^{t+k}=C^{k}Y^{t}.$$ (8)

$$Y^{t-k}=D^{k}Y^{t}.$$ (9)

$${\hat{F}}^{t\pm k}=\chi_{d}\left(Y^{t\pm k}\right).$$ (10)

where $Y^{t+k}$ and $Y^{t-k}$ represent the low-dimensional state after k steps of forward and backward prediction, respectively. ${\hat{F}}^{t\pm k}$ represents the predictive high-dimensional nonlinear state.

Equations 11, 12 define the loss function of forward prediction ( $L_{fwd}$ ) and backward prediction ( $L_{bwd}$ ) to minimize the difference between the high-dimensional predictive value and true states at each step, respectively.

$$L_{\text{fwd}}=\frac{1}{k}\sum_{s=1}^{k}{\left|\left|{\hat{F}}^{t+s}-F^{t+s}\right|\right|}_{\text{MSE}}.$$ (11)

$$L_{\text{bwd}}=\frac{1}{k}\sum_{s=1}^{k}{\left|\left|{\hat{F}}^{t-s}-F^{t-s}\right|\right|}_{\text{MSE}}.$$ (12)

Equation 13 defines the loss function ( $L_{idy}$ ) to minimize the difference between the predictive low-dimensional state obtained by the C and D matrices and defines such a low-dimensional state that is mapped from the true high-dimensional state by mapping $\chi_{e}$ .

$$L_{\text{idy}}=\frac{1}{k}\overset{k}{\underset{s=1}{\sum }}\left[\|C^{s}\chi_{e}\left(F^{t}\right)-\chi_{e}{\left(F^{t+s}\right)\|}_{\text{MSE}}+\|D^{s}\chi_{e}\left(F^{t}\right)-\chi_{e}{\left(F^{t-s}\right)\|}_{\text{MSE}}\right].$$ (13)

Additionally, we employ loss function ( $L_{con}$ ) by Eq. 14 to train the parameters $a_{i}$ and $b_{i}$ in the matrices C and D , respectively.

$$L_{\text{con}}=\frac{1}{k}\sum_{s=1}^{k}\left[{\left\|\chi_{d}\left(D^{s}C^{s}Y^{t}\right)-F^{t}\right\|}_{\text{MSE}}+{\left\|\chi_{d}\left(C^{s}D^{s}Y^{t}\right)-F^{t}\right\|}_{\text{MSE}}\right]$$ (14)

### Parameter Estimation for the EKATP

Equation 15 optimizes the key parameters for the EKATP by minimizing L .

$$L=\lambda_{id}L_{id}+\lambda_{fwd}L_{fwd}+\lambda_{bwd}L_{bwd}+\lambda_{idy}L_{idy}+\lambda_{con}L_{con}.$$ (15)

Here, $\lambda_{id}$ , $\lambda_{\text{fwd}}$ , $\lambda_{\text{bwd}}$ , $\lambda_{\text{idy}}$ and $\lambda_{\text{con}}$ are user-defined hyperparameters.

## Experiments

This section evaluates the predictability of the proposed EKATP for high-dimensional nonlinear multi-omics datasets by comparing it with recurrent neural networks (RNNs) (Jiang and Lai, 2019), long short-term memory (LSTM) (Hochreiter and Schmidhuber, 1997), dynamic Autoencoder (DAE) (Lusch et al., 2018) and Koopman Autoencoder (KAE) (Azencot et al., 2020). The detailed experimental setup is listed in Supplementary Presentation S2. In addition, we detail the workflow chart and list the related pseudocode in Supplementary Figure S1; Supplementary Presentation S3.

### Genomics

We usually employ the chaotic Lorentz system (Lorenz, 1963) to describe a gene expression time series with a low-dimensional manifold (Sauer et al., 1991) by Eq. 16,

$$\left\{ \begin{array}{l} x_{t+1}=x_{t}+h\left(\eta \left(y_{t}-z_{t}\right)\right)\\ y_{t+1}=y_{t}+h\left(x_{t}\left(\rho -z_{t}\right)-y_{t}\right)\\ z_{t+1}=z_{t}+h\left(x_{t}y_{t}-\beta z_{t}\right) \end{array}\right.,$$ (16)

where η and ρ represent the Prandtl and Rayleigh numbers, respectively. β is related to geometry, and t represents time. h represents the level of the complicated nonlinear system. When h is greater, the nonlinear relationship between genes becomes more complicated.

Since gene expression time series contains considerable noise, we employ white Gaussian noise (Li et al., 2017) to simulate the noise by Eq. 17,

$$\{\begin{array}{c} \tilde{x}=x+\varepsilon_{x}\\ \tilde{y}=y+\varepsilon_{y}\\ \begin{array}{c} \tilde{z}=z+\varepsilon_{z} \end{array} \end{array}.$$ (17)

where $\tilde{x}$ , $\tilde{y}$ and $\tilde{z}$ represent data with noise. $\varepsilon_{x}$ , $\varepsilon_{y}$ and $\varepsilon_{z}$ represent the white Gaussian noise for x , y and z by normal distributions $N\left(0,\sigma^{2}\right)$ with a zero mean and a standard deviation σ . The standard deviation σ is referred to as the noise intensity.

Here, we describe how to obtain a high-dimensional gene expression time series with a low-dimensional manifold as follows. First, we generate the three-dimensional time series $V=\left(V^{1},V^{2},\ldots ,V^{T}\right)\in {\mathbb{R}}^{3}$ ( T is the total step), which is listed in Supplementary Tables S1.1, S1.2, S1.3. Next, we develop a random orthogonal transformation (Anderson et al., 1987) matrix $P\in {\mathbb{R}}^{96\times 3}$ . Finally, we map the state of a 3-dimensional time series onto the state of a 96-dimensional time series by Eq. 18 to simulate a high-dimensional gene expression time series $F=\left(F^{1},F^{2},\ldots ,F^{T}\right)\in {\mathbb{R}}^{96}$ with a 3-dimensional manifold, which is listed in Supplementary Tables S1.4, S1.5, S1.6.

F=PV. (18)

To prove the accuracy and robustness of the EKATP, we generate a small-scale system containing T = 1,050 steps and choose the last 50 steps to visualize the predictive power of the EKATP.

**FIGURE 2:**
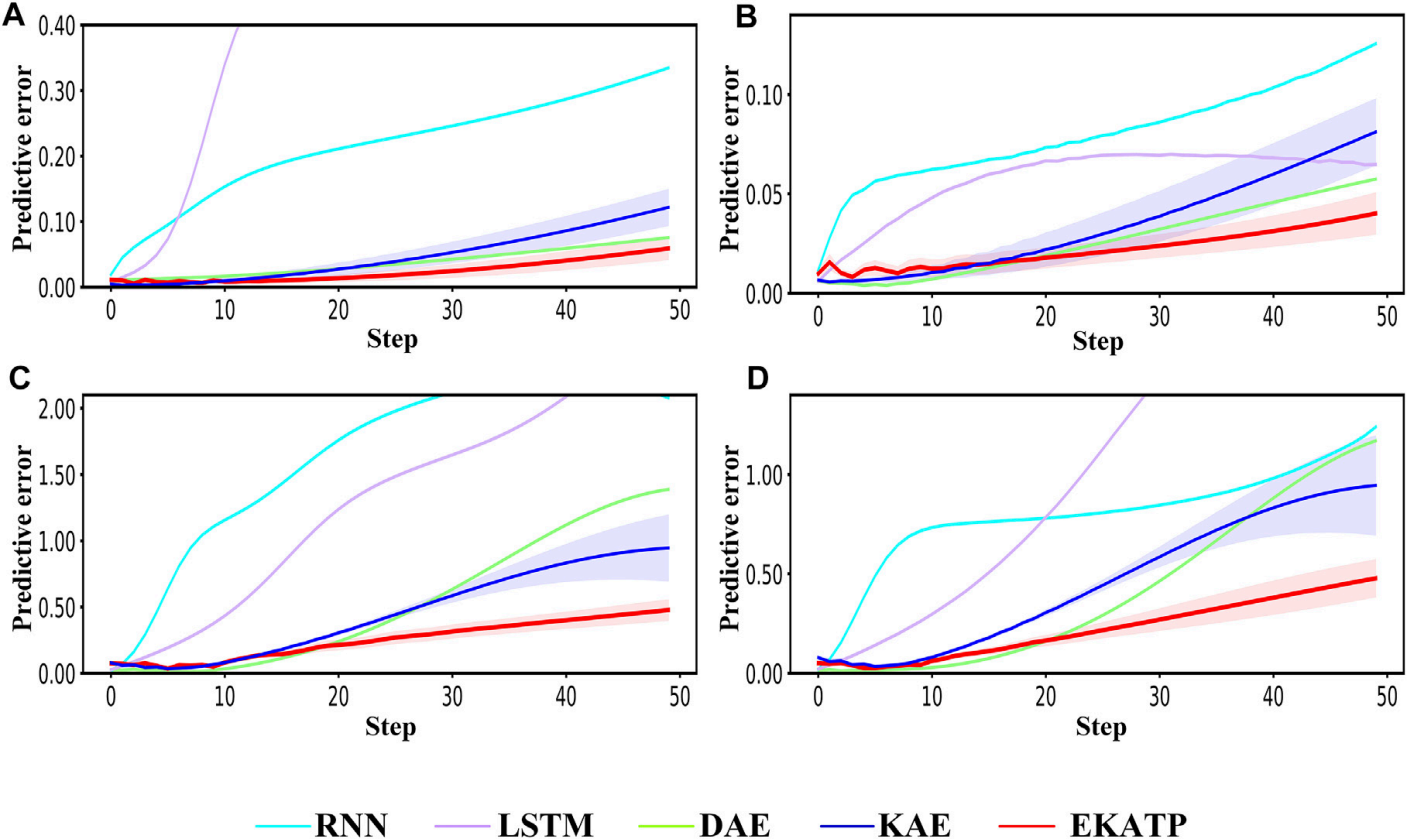
Comparison among the RNN, LSTM, DAE, KAE and EKATP. The abscissa represents the step, and the ordinate represents the predictive error. **(A)** The initial conditions are h = 0.003 and σ = 0.00. **(B)** The initial conditions are h = 0.003 and σ = 0.01. **(C)** The initial conditions are h = 0.006 and σ = 0.00. **(D)** The initial conditions are h = 0.006 and σ = 0.01.

Figure 2 shows the predictive error in the range of 50 steps under different initial conditions and environments. Detailed information is listed in Supplementary Tables S1.7, S1.8; Supplementary Presentation S4.

Figures 2A,C demonstrates that the EKATP not only has less of a predictive error than the existing methods under a clean environment ( σ =0.00) but also has a stable predictive error when the complexity h increases from 0.003 to 0.006. In particular, with the increase in predictive steps, the predictive error of the EKATP increases slower than that of the existing methods.

Figures 2B,D shows that the EKATP not only has less of a predictive error than previous methods under a noisy environment ( σ =0.01) but also has a predictive error that slightly fluctuates when h increases from 0.003 to 0.006. Moreover, after 25 steps, the predictive error of the EKATP increases much slower than that of the existing methods.

Figure 2 indicates that the EKATP has greater predictive accuracy and robustness than excitation methods in clean and noisy environments.

To further prove the generalizability of the EKATP, we generate a large-scale system containing T = 15,000 steps under the condition of h = 0.003 and σ = 0.00. After that, we randomly choose three different time periods to train and test the model as follows, the procedure of which is detailed in Supplementary Table S1.9.

First, since the 3-dimensional time series state and 96-dimensional time series state are diffeomorphic (Sauer et al., 1991), which is indicated by the data preprocessing procedure, it implies that the mapping between these two time series is reversible. Here, we map the 96-dimensional gene expression predictive results onto a 3-dimensional space by orthogonal inverse transformation (Anderson et al., 1987) to visualize the predictive result of the EKATP.

**FIGURE 3:**
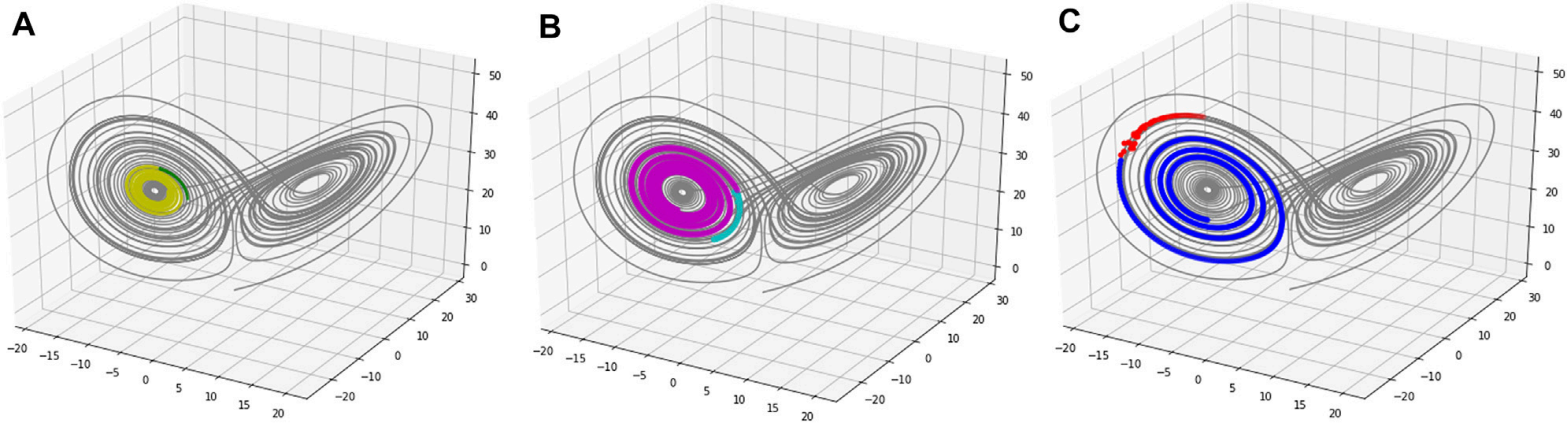
The 50-step predictive trajectories of the EKATP are under initial conditions h = 0.003 and σ = 0.00. Grey colors represent full true data. **(A)** This is the predictive situation of the first period. Yellow and green colors represent true and predictive data, respectively. **(B)** This is the predictive situation of the second period. Purple and cyan colors represent true and predictive data, respectively. **(C)** This is the predictive situation of the third period. Blue and red colors represent true and predictive data, respectively.

Figures 3A,B,C demonstrates that the predictive results of the EKATP are close to the true value for different periods of a time series. Figure 3 shows that the EKATP can accurately predict the gene expression time series at different periods, implying that it has a strong generalizability, even in a very complicated nonlinear environment.

### Proteomics

We always use a nonlinear pendulum model (Hirsch, 1974) with oscillatory behaviour to describe a proteomics time series with a low-dimensional manifold (Sauer et al., 1991) by Eq. 19,

$$\left\{ \begin{array}{l} \frac{d^{2}\theta }{dt^{2}}+\frac{g}{l}\sin\theta =0\\ \theta \left(t_{0}\right)=h \end{array}\right..$$ (19)

where  l , g and t denote the length, gravity and time, respectively. h denotes the initial value of θ , which represents the level of the complicated nonlinear system. When h is greater, the nonlinear relationship between proteins becomes more complicated.

Since a considerable amount of noise exists in a protein time series, we employ white Gaussian noise (Li et al., 2017) to describe it by Eq. 20,

$$\left\{ \begin{array}{l} \tilde{\theta }=\theta +\varepsilon_{\theta }\\ \tilde{\dot{\theta }}=\dot{\theta }+\varepsilon_{\dot{\text{θ}}} \end{array}\right.,$$ (20)

where $\tilde{\theta }$ and $\tilde{\dot{\theta }}$ represent data with noise. $\varepsilon_{\theta }$ and $\varepsilon_{\dot{\text{θ}}}$ represent the noise Gaussian terms for θ and $\dot{\theta }$ by normal distributions $N\left(0,\sigma^{2}\right)$ with a zero mean and a standard deviation σ .

Here, we describe how to obtain a high-dimensional proteomics time series with a low-dimensional manifold. First, we generate the 2-dimensional time series $V=\left(V^{1},V^{2},\ldots ,V^{T}\right)\in {\mathbb{R}}^{2}$ , which is listed in Supplementary Tables S2.1, S2.2. Next, we develop a random orthogonal transformation (Anderson et al., 1987) matrix $P\in {\mathbb{R}}^{64\times 2}$ . Finally, we map the state of a 2-dimensional time series onto the state of a 64-dimensional time series by Eq. 18 to simulate a high-dimensional proteomics time series $F=\left(F^{1},F^{2},\ldots ,F^{T}\right)\in {\mathbb{R}}^{64}$ with a 2-dimensional manifold, which is listed in Supplementary Tables S2.3, S2.4.

To prove the accuracy and robustness of the EKATP, we generate a system containing T = 1,600 steps and choose the last 1,000 steps to visualize the predictability for the EKATP.

**FIGURE 4:**
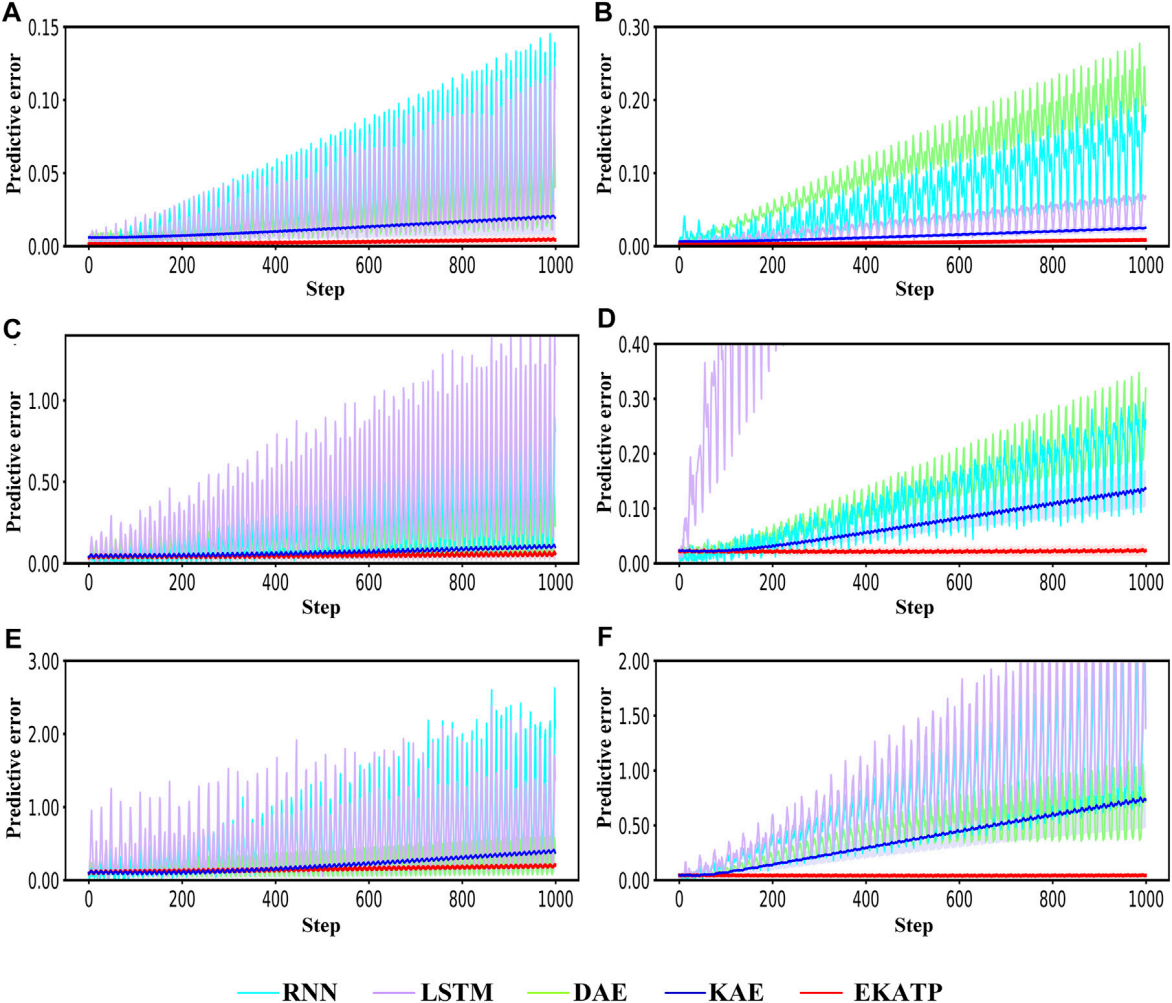
Comparison with the RNN, LSTM, DAE, KAE and EKATP. The abscissa represents the step, and the ordinate represents the predictive error. **(A)** The initial conditions are h = 0.8 and σ = 0.00. **(B)** The initial conditions are h = 2.4 and σ = 0.00. **(C)** The initial conditions are h = 0.8 and σ = 0.03. **(D)** The initial conditions are h = 2.4 and σ = 0.03. **(E)** The initial conditions are h = 0.8 and σ = 0.08. **(F)** The initial conditions are h = 2.4 and σ = 0.08.

Figure 4 shows that the EKATP can effectively predict a proteomic time series under clean and noisy environments within 1,000 steps, the details of which are listed in Supplementary Tables S2.5, S2.6; Supplementary Presentation S4.

Figures 4A,B shows that the EKATP not only has less of a predictive error under a clean environment ( σ =0.00) than the existing methods but also maintains a smaller predictive error when h increases from 0.8 to 2.4. Moreover, the predictive error of the EKATP increases much slower than that of the existing methods when the predictive step increases.

Figures 4C,D demonstrates that the EKATP has less of a predictive error under a noise environment ( σ =0.03) than the existing methods. When h increases from 0.8 to 2.4, the predictive error of the EKATP remains stable. In particular, with the increase in predictive steps, the predictive error of the EKATP increases much slower than that of the existing methods.

Figures 4E,F indicates that the EKATP not only has less of a predictive error under a noise environment ( σ =0.08) than the existing methods but also has a predictive error of the EKATP that remains stable when h increases from 0.8 to 2.4. In particular, when the predictive steps are long enough (after 500 steps), the predictive error of previous methods increases much faster than that of the EKATP.

Figures 4A,C,E shows that the predictive error of the EKATP remains stable when the noise intensity σ increases from 0 to 0.08 under complexity h = 0.8. Figures 4B,D,F shows that the predictive error of the EKATP remains stable when the noise intensity σ increases from 0 to 0.08 under complexity h = 2.4.

Figure 4 demonstrates that the predictive accuracy and robustness of the EKATP outperforms the existing methods under clean and noisy environments.

Since Figure 4 shows that KAE has a better predictive effect than the other existing methods, we use it to compare the predictive performance with the EKATP by visualizing the predictive trajectory.

**FIGURE 5:**
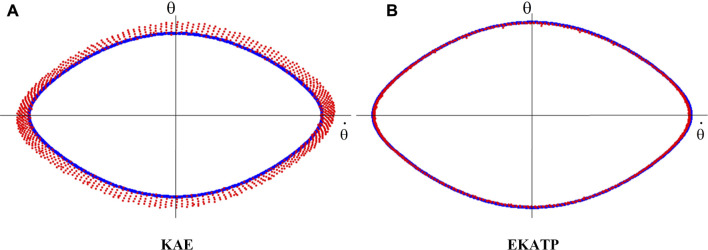
The prediction trajectories within 1,000 steps under the initial conditions of h = 2.4 and σ = 0.03. The abscissa represents $\dot{\theta }$ , the ordinate represents θ , blue colours represent true data and red dots represent predictive data. **(A)** KAE. **(B)** EKATP.

Indicated by our data preprocessing procedure, since the 2-dimensional time series state and 64-dimensional time series state are diffeomorphic (Sauer et al., 1991), the mapping between these two time series is reversible. Here, we map the 64-dimensional protein time series predictive results onto a 2-dimensional space by orthogonal inverse transformation (Anderson et al., 1987) to visualize the predictive time series trajectory. Figure 5 shows the predictive trajectories of the KAE and EKATP within 1,000 steps under the initial conditions of h = 2.4 and σ = 0.03, which show that the predictive protein time series trajectory of the EKATP (Figure 5B) is much closer to the true trajectory than that of the KAE (Figure 5A). Figure 5 further indicates that the predictive accuracy and robustness of the EKATP is better than that of the KAE.

To further prove that the EKATP has strong generalizability, we randomly selected 20 pieces of different protein time series data for model training and analysis. The details are listed in Table 1; Supplementary Table S2.7.

**TABLE 1:** Predictive error at 1,000 steps for both the KAE and EKATP.

Model	h	θ	Predictive error				*p*-Value
			Min	Max	Avg	Var	
KAE	0.8	0.00	0.427	0.012	0.052	8.29e−03	2.60e−02
EKATP	0.8	0.00	**0.001**	**0.006**	**0.003**	**2.18e−06**	
KAE	0.8	0.03	0.038	0.253	0.112	2.80e−03	2.11e−04
EKATP	0.8	0.03	0.038	**0.089**	**0.058**	**1.42e−04**	
KAE	2.4	0.00	0.020	0.225	0.067	2.03e−03	5.79e−06
EKATP	2.4	0.00	**0.003**	**0.010**	**0.005**	**4.20e−06**	
KAE	2.4	0.03	0.030	0.967	0.131	4.04e−02	2.42e−02
EKATP	2.4	0.03	**0.011**	**0.040**	**0.021**	**5.79e−05**	

After we employ 20 different proteomics time series datasets to test the KAE and EKATP, Table 1 shows the predictive error of the KAE and EKATP at 1,000 steps under different initial noise and complexity ( h ) conditions, which demonstrates that the EKATP has less of a statistically significant minimum, maximum, average and variance of the predictive error than the KAE under each noise and complexity ( h ) condition (*p*-value <0.05) (Gao et al., 2017; Li et al., 2017; Gao et al., 2021). Table 1 implies that the EKATP has statistically significant predictive power for different time series datasets.

### Metabolomics

We usually employ a nonlinear biological fluid system (Noack et al., 2003) to describe the high-dimensional metabolic time series with a low-dimensional manifold (Sauer et al., 1991) for the flow behavior of biological fluids simulation by Eq. 21,

$$\left\{ \begin{array}{l} \dot{x}=\gamma x-\omega y+Axz\\ \dot{y}=\omega x+\gamma y+Ayz\\ \dot{z}=-\lambda \left(z-x^{2}-y^{2}\right) \end{array}\right.,$$ (21)

where γ , ω and A determine the size of the fluid. λ determines the speed of the dynamics of z . The different initial values of x , y and z determine the different nonlinear complexities of the metabolomics time series. We use the initial conditions $\zeta_{1}$ ( x =0, y = -0.01,  z = 0) and $\zeta_{2}$ ( x =0.01, y = -0.1,  z = 0.5) to generate a high-dimensional metabolomics time series with low complexity $h_{1}$ and high complexity $h_{2}$ , respectively.

Since the metabolomics time series contains considerable noise, we employ white Gaussian noise (Li et al., 2017) to describe it by Eq. 22,

$$\{\begin{array}{c} \tilde{x}=x+\varepsilon_{x}\\ \tilde{y}=y+\varepsilon_{y}\\ \begin{array}{c} \tilde{z}=z+\varepsilon_{z} \end{array} \end{array}.$$ (22)

where $\tilde{x}$ , $\tilde{y}$ and $\tilde{z}$ represent data with noise. $\varepsilon_{x}$ , $\varepsilon_{y}$ and $\varepsilon_{z}$ represent the white Gaussian noise for x , y and z by normal distributions $N\left(0,\sigma^{2}\right)$ with a zero mean and a standard deviation σ .

**FIGURE 6:**
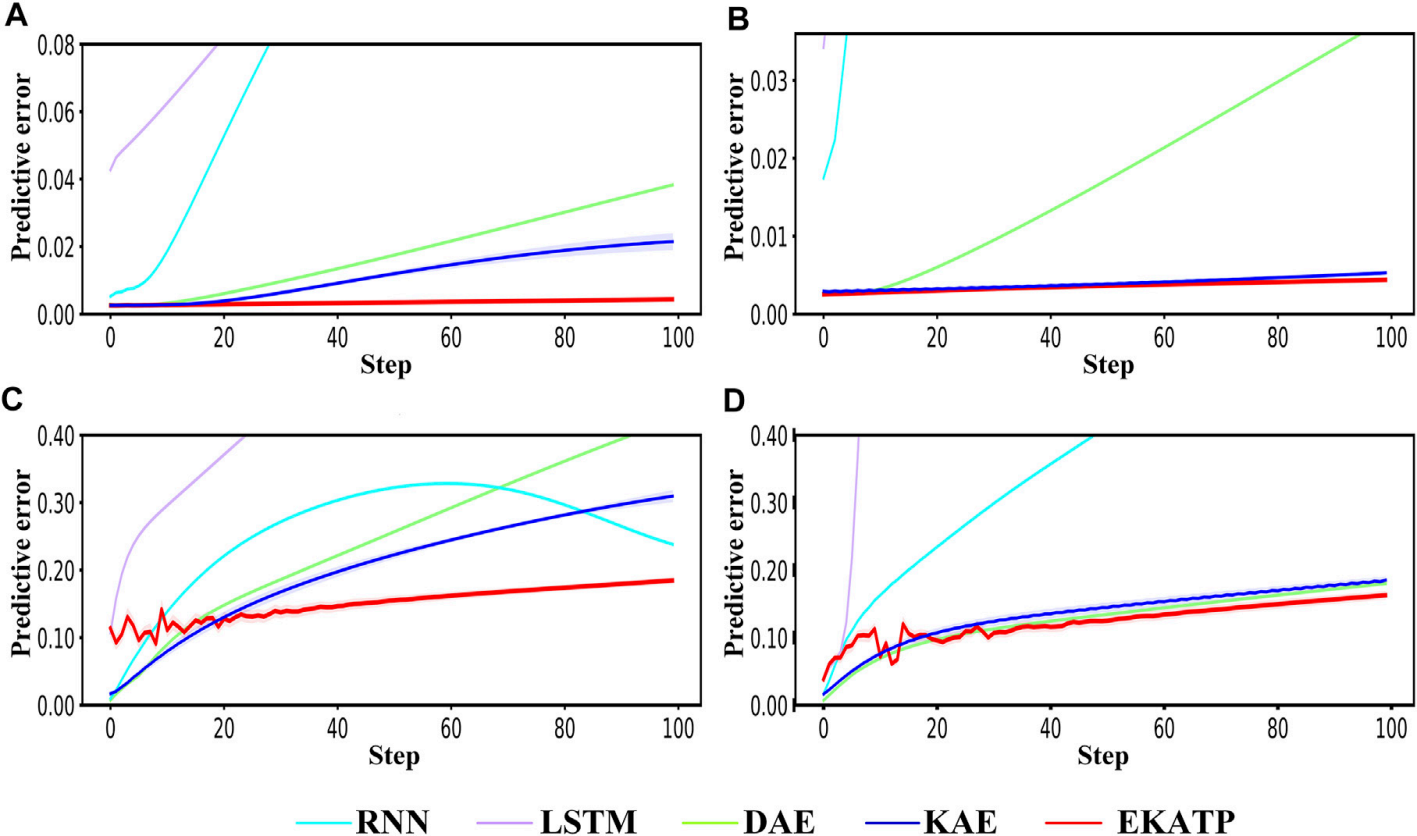
Comparison of the RNN, LSTM, DAE, KAE and EKATP. The abscissa represents the time step, and the ordinate represents the predictive error. **(A)** The initial conditions are $h_{1}$ and σ = 0.000. **(B)** The initial conditions are $h_{1}$ and σ = 0.001. **(C)** The initial conditions are $h_{2}$ and σ = 0.000. **(D)** The initial conditions are $h_{2}$ and σ = 0.001.

Fig. 6 Comparison of the RNN, LSTM, DAE, KAE and EKATP. The abscissa represents the time step, and the ordinate represents the predictive error. (A) The initial conditions are $h_{1}$ and σ = 0.000. (B) The initial conditions are $h_{1}$ and σ = 0.001. (C) The initial conditions are $h_{2}$ and σ = 0.000. (D) The initial conditions are $h_{2}$ and σ = 0.001.

Here, we show how to generate a high-dimensional metabolomics time series with a low-dimensional manifold. First, we build up the 3-dimensional time series $V=\left(V^{1},V^{2},\ldots ,V^{T}\right)\in {\mathbb{R}}^{3}$ , which is listed in Supplementary Tables S3.1; S3.2. Next, we develop a random orthogonal transformation (Anderson et al., 1987) matrix $P\in {\mathbb{R}}^{96\times 3}$ . Finally, we map the state of the 3-dimensional time series onto the state of the 96-dimensional time series by Eq. 18 to simulate a high-dimensional metabolic time series $F=\left(F^{1},F^{2},\ldots ,F^{T}\right)\in {\mathbb{R}}^{96}$ with the 3-dimensional manifold, which is listed in Supplementary Tables S3.3, S3.4.

To demonstrate the accuracy and robustness of the EKATP, we generate a system containing T = 900 steps and choose the last 100 steps to visualize the predictive result of the EKATP. Figure 6 shows the predictive results of the metabolic time series under different initial conditions and environments for the last 100 steps. Detailed information is listed in Supplementary Tables S3.5, S3.6; Supplementary Presentation S4.

Figures 6A,C demonstrates that the EKATP has less of a predictive error under a clean environment ( σ =0.000) than the existing methods. When the complexity of h increases, the predictive error of the EKATP remains stable. With the increase in the predictive step, the predictive error of the existing methods increases rapidly, while the predictive error of the EKATP remains small.

Figures 6B,D suggests that the EKATP not only has less of a predictive error under a low noise intensity environment ( σ =0.001) than the existing methods but also has a predictive error of the EKATP that remains stable when h increases. In particular, when the predictive steps are long enough, the predictive error of the EKATP increases much slower than that of the existing methods.

Figure 6 implies that the EKATP has better predictive accuracy and robustness than the existing methods under clean and weakly noisy environments.

**FIGURE 7:**
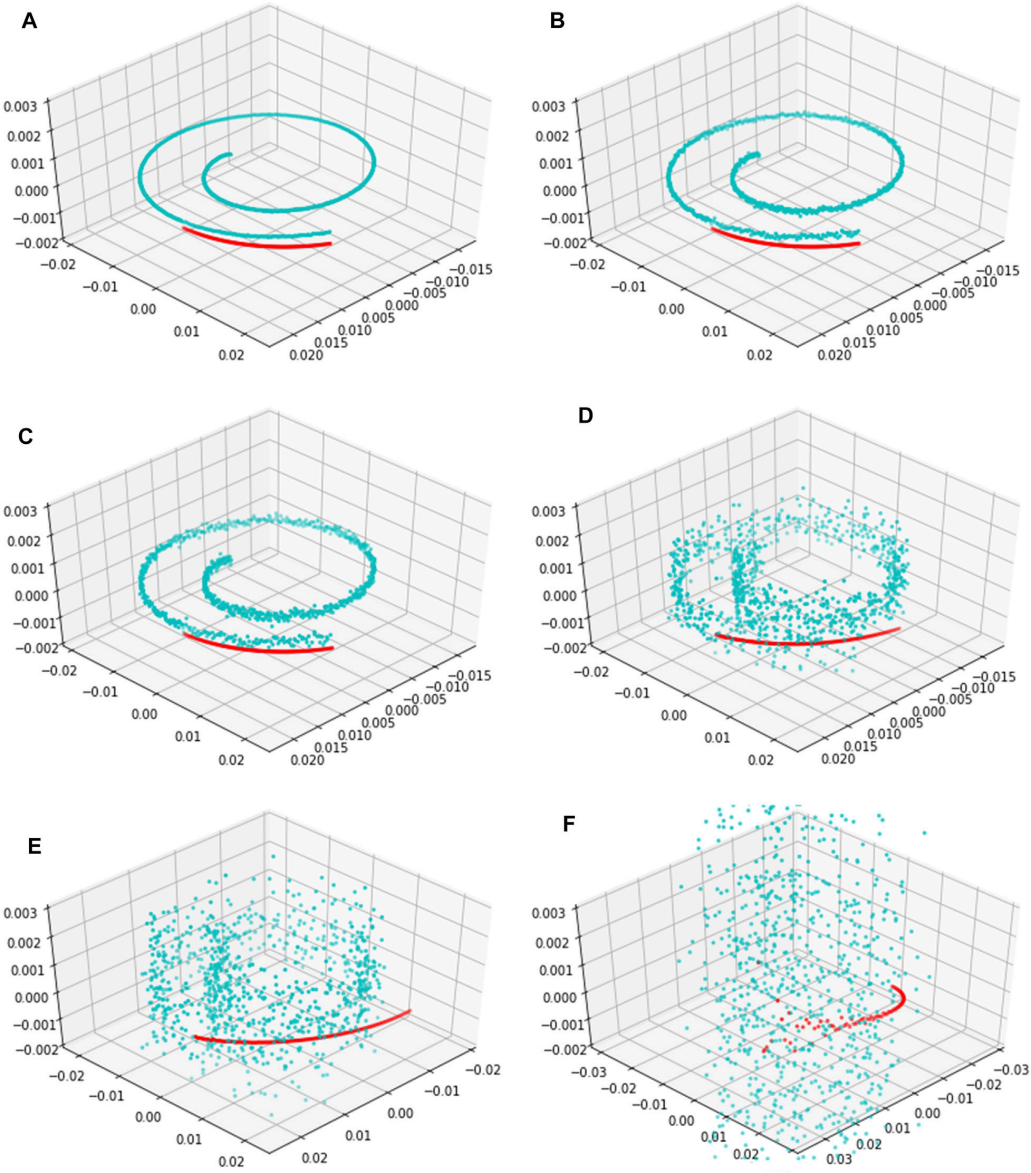
The predictive trajectories of the EKATP within 100 steps under different intensities of noise. Cyan dots represent true data with an interval of t∈ [0:900]. Red dots represent predictive data with an interval of t∈ [800:900]. **(A)** σ = 0.001. **(B)** σ = 0.005. **(C)** σ = 0.010. **(D)** σ = 0.050. **(E)** σ = 0.100. **(F)** σ = 0.500.

Since a metabolomics time series usually has strong noise intensity (Mak et al., 2015), we use the EKATP to predict a high-dimensional metabolomics time series under strong noise intensities to prove its robustness. Because the 3-dimensional time series state and the 96-dimensional time series state are diffeomorphic (Sauer et al., 1991), the mapping between these two time series is reversible. Thus, after we map the 96-dimensional metabolic time series predictive results onto a 3-dimensional space by orthogonal inverse transformation (Anderson et al., 1987), Figure 7 shows the predictive time series trajectories by the EKATP under different intensities of noise. We select the last 100 steps to validate the predictive power of the EKATP as in the previous setup (Supplementary Table S3.6). The results demonstrate that although the true data become gradually messy when we increase the noise intensity σ , the predictive time series trajectory of the EKATP is still very close to the true data to a certain extent (Figures 7A,B,C,D,E,F), which implies that the EKATP still has a satisfactory predictive performance when we increase the noise intensity.

Moreover, we use Eqs 23, 24 to calculate the Pearson correlation coefficient (PCC) (Abar et al., 2017) and the root mean squared error (RMSE) (Abar et al., 2017) between predictive and true data under different noise intensities.

Here, $V^{t}$ and ${\hat{V}}^{t}$ represent the true and predictive data at time t . μ and $\hat{\mu }$ represent the average value for true and predictive data, respectively. p represents the predictive step size.

$$\text{PCC}=\frac{\sum_{t=1}^{t=p}\left({\hat{V}}^{t}-\hat{\mu }\right)\left(V^{t}-\mu \right)}{\sqrt{\sum_{t=1}^{t=p}{\left({\hat{V}}^{t}-\hat{\mu }\right)}^{2}}\sqrt{\sum_{t=1}^{t=p}{\left(V^{t}-\mu \right)}^{2}}}.$$ (23)

RMSE=1p∑t=1t=p||V^t−Vt||2. (24)

**FIGURE 8:**
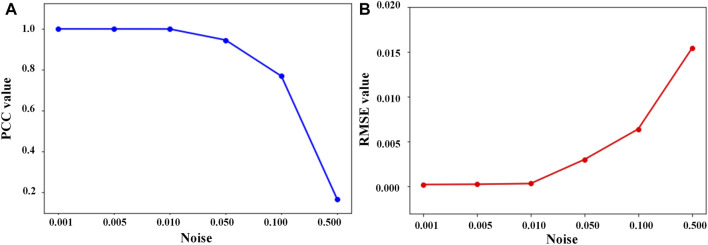
PCC and RMSE values between predictive and true data under different noise intensities. The details are listed in Supplementary Table S3.7. **(A)** PCC value between predictive and true data, where the abscissa represents the noise intensity and the ordinate represents the PCC value. **(B)** RMSE value between predictive and true data, where the abscissa represents the noise intensity and the ordinate represents the RMSE value.

Figure 8A shows that the PCC value of the EKATP gradually decreases when we increase the noise intensity σ , but the overall value is relatively high. Figure 8B indicates that with the increase in noise intensity σ , although the RMSE value of the EKATP gradually increases, it is still relatively small. Thus, we conclude that the EKATP can effectively avoid noise interference and is robust enough under a very strong noise intensity condition.

## Conclusion and Future Work

To answer the three proposed questions, this study developed an EKATP to predict the future state of a high-dimensional nonlinear multi-omics time series. First, we select key features from high-dimensional nonlinear multi-omics time series data. After that, we map these key features to the low-dimensional linear space. Next, we obtain the future state of the multi-omics time series by learning the evolutionary relationship between the adjacent states of the time series in the low-dimensional linear space. Finally, we predict the future state of the high-dimensional nonlinear multi-omics time series by mapping the low-dimensional linear predictive state back to the high-dimensional nonlinear space. The experimental results demonstrate that the EKATP can greatly improve the accuracy, robustness and generalisability to predict the future state of a time series for genomics (Figures 2, 3), proteomics (Figures 4, 5; Table 1) and metabolomics (Figures 6–8) datasets.

However, there are still several shortcomings to the current study. For example, we are still unclear on the impact of embedding dimensions from high-dimensional nonlinear space to low-dimensional linear space on predictive accuracy and the way to use high-performance computing to increase the efficiency of the EKATP. Applying the EKATP to network biological datasets (Liu X. et al., 2020) is also the direction we need to continue the study. Thus, we will improve the EKATP from these perspectives in the distant future.

## Data Availability

The original contributions presented in the study are included in the article/Supplementary Material, further inquiries can be directed to the corresponding author.

## References

[B1] Abar T. El Asmi A. S. Asmi S. E. (2017). “Machine Learning Based QoE Prediction in SDN Networks,” in Proceedings of the 2017 13th International Wireless Communications and Mobile Computing Conference (IWCMC), Valencia, Spain, June 2017. 10.1109/IWCMC.2017.7986488

[B2] Anderson T. W. Olkin I. Underhill L. G. (1987). Generation of Random Orthogonal Matrices. SIAM J. Sci. Stat. Comput. 8 (4), 625–629. 10.1137/0908055

[B3] Azencot O. Erichson N. B. Lin V. Mahoney M. (2020). “Forecasting Sequential Data Using Consistent Koopman Autoencoders,” in Proceedings of the 37th International Conference on Machine Learning, 475–485.

[B4] Bianconi F. Antonini C. Tomassoni L. Valigi P. (2020). Robust Calibration of High Dimension Nonlinear Dynamical Models for Omics Data: An Application in Cancer Systems Biology. IEEE Trans. Contr. Syst. Technol. 28 (1), 196–207. 10.1109/TCST.2018.2844362

[B5] Chen P. Liu R. Aihara K. Chen L. (2020). Autoreservoir Computing for Multistep Ahead Prediction Based on the Spatiotemporal Information Transformation. Nat. Commun. 11 (1), 4568. 10.1038/s41467-020-18381-0 32917894 PMC7486927

[B6] Davidson E. Levin M. (2005). Gene Regulatory Networks. Proc. Natl. Acad. Sci. 102 (14), 4935. 10.1073/pnas.0502024102 15809445 PMC556010

[B7] Eisenhammer T. Hübler A. Packard N. Kelso J. A. S. (1991). Modeling Experimental Time Series with Ordinary Differential Equations. Biol. Cybern. 65 (2), 107–112. 10.1007/BF00202385 1912002

[B8] Fischer H. P. (2008). Mathematical Modeling of Complex Biological Systems: from Parts Lists to Understanding Systems Behavior. Alcohol. Res. Health 31 (1), 49–59. 23584751 PMC3860444

[B9] Gao H. Yin Z. Cao Z. Zhang L. (2017). Developing an Agent-Based Drug Model to Investigate the Synergistic Effects of Drug Combinations. Molecules 22 (12), 2209. 10.3390/molecules22122209 PMC6149923 29240712

[B10] Gao J. Liu P. Liu G.-D. Zhang L. (2021). Robust Needle Localization and Enhancement Algorithm for Ultrasound by Deep Learning and Beam Steering Methods. J. Comput. Sci. Technol. 36 (2), 334–346. 10.1007/s11390-021-0861-7

[B11] Hinton G. E. Salakhutdinov R. R. (2006). Reducing the Dimensionality of Data with Neural Networks. Science 313 (5786), 504–507. 10.1126/science.1127647 16873662

[B12] Hirsch M. (1974). Differential Equations, Dynamical Systems, and Linear Algebra Mathematics. America: Academic press. 10.1016/s0079-8169(08)x6044-1

[B13] Hochreiter S. Schmidhuber J. (1997). Long Short-Term Memory. Neural Comput. 9 (8), 1735–1780. 10.1162/neco.1997.9.8.1735 9377276

[B14] Holmes P. Lumley J. L. Berkooz G. Rowley C. W. (2012). Turbulence, Coherent Structures, Dynamical Systems and Symmetry. Cambridge, UK, Cambridge University Press.

[B15] Iuchi H. Sugimoto M. Tomita M. (2018). MICOP: Maximal Information Coefficient-Based Oscillation Prediction to Detect Biological Rhythms in Proteomics Data. BMC Bioinformatics 19 (1), 249. 10.1186/s12859-018-2257-4 29954316 PMC6025708

[B16] Ji Z. Yan K. Li W. Hu H. Zhu X. (2017). Mathematical and Computational Modeling in Complex Biological Systems. Biomed. Res. Int. 2017, 1–16. 10.1155/2017/5958321 PMC5366773 28386558

[B17] Jiang J. Lai Y.-C. (2019). Model-free Prediction of Spatiotemporal Dynamical Systems with Recurrent Neural Networks: Role of Network Spectral Radius. Phys. Rev. Res. 1 (3), 033056. 10.1103/PhysRevResearch.1.033056

[B18] Koopman B. O. (1931). Hamiltonian Systems and Transformation in Hilbert Space. Proc. Natl. Acad. Sci. 17 (5), 315–318. 10.1073/pnas.17.5.315 16577368 PMC1076052

[B19] Lai Q. Zhao X.-W. Huang J.-N. Pham V.-T. Rajagopal K. (2018). Monostability, Bistability, Periodicity and Chaos in Gene Regulatory Network. Eur. Phys. J. Spec. Top. 227 (7), 719–730. 10.1140/epjst/e2018-700132-8

[B20] Levnajić Z. Tadić B. (2010). Stability and Chaos in Coupled Two-Dimensional Maps on Gene Regulatory Network of Bacterium *E. coli* . Chaos 20 (3), 033115. 10.1063/1.3474906 20887055

[B21] Li T. Cheng Z. Zhang L. (2017). Developing a Novel Parameter Estimation Method for Agent-Based Model in Immune System Simulation under the Framework of History Matching: A Case Study on Influenza A Virus Infection. Ijms 18 (12), 2592. 10.3390/ijms18122592 PMC5751195 29194393

[B22] Liang Y. Kelemen A. (2017a). Computational Dynamic Approaches for Temporal Omics Data with Applications to Systems Medicine. BioData Mining 10 (1), 20. 10.1186/s13040-017-0140-x 28638442 PMC5473988

[B23] Liang Y. Kelemen A. (2017b). Dynamic Modeling and Network Approaches for Omics Time Course Data: Overview of Computational Approaches and Applications. Brief. Bioinform. 19 (5), 1051–1068. 10.1093/bib/bbx036 28430854

[B24] Liu G.-D. Li Y.-C. Zhang W. Zhang L. (2020). A Brief Review of Artificial Intelligence Applications and Algorithms for Psychiatric Disorders. Engineering 6 (4), 462–467. 10.1016/j.eng.2019.06.008

[B25] Liu X. Maiorino E. Halu A. Glass K. Prasad R. B. Loscalzo J. (2020). Robustness and Lethality in Multilayer Biological Molecular Networks. Nat. Commun. 11 (1), 6043. 10.1038/s41467-020-19841-3 33247151 PMC7699651

[B26] Lockhart D. J. Winzeler E. A. (2000). Genomics, Gene Expression and DNA Arrays. Nature 405 (6788), 827–836. 10.1038/35015701 10866209

[B27] Lorenz E. N. (1963). Deterministic Nonperiodic Flow. J. Atmos. Sci. 20 (2), 130–141. 10.1175/1520-0469(1963)020<0130:dnf>2.0.co;2

[B28] Lusch B. Kutz J. N. Brunton S. L. (2018). Deep Learning for Universal Linear Embeddings of Nonlinear Dynamics. Nat. Commun. 9 (1), 4950. 10.1038/s41467-018-07210-0 30470743 PMC6251871

[B29] Mak T. D. Laiakis E. C. Goudarzi M. Fornace A. J. Jr. (2015). Selective Paired Ion Contrast Analysis: a Novel Algorithm for Analyzing Postprocessed LC-MS Metabolomics Data Possessing High Experimental Noise. Anal. Chem. 87 (6), 3177–3186. 10.1021/ac504012a 25683158 PMC4519008

[B30] Mann M. Hendrickson R. C. Pandey A. (2001). Analysis of Proteins and Proteomes by Mass Spectrometry. Annu. Rev. Biochem. 70 (1), 437–473. 10.1146/annurev.biochem.70.1.437 11395414

[B31] Noack B. R. Afanasiev K. Morzyński M. Tadmor G. Thiele F. (2003). A Hierarchy of Low-Dimensional Models for the Transient and post-transient cylinder Wake. J. Fluid Mech. 497, 335–363. 10.1017/S0022112003006694

[B32] Perez-Riverol Y. Kuhn M. Vizcaíno J. A. Hitz M.-P. Audain E. (2017). Accurate and Fast Feature Selection Workflow for High-Dimensional Omics Data. PloS one 12 (12), e0189875. 10.1371/journal.pone.0189875 29261781 PMC5738110

[B33] Sauer T. Yorke J. A. Casdagli M. (1991). Embedology. J. Stat. Phys. 65 (3), 579–616. 10.1007/BF01053745

[B34] Sevim V. Rikvold P. A. (2008). Chaotic Gene Regulatory Networks Can Be Robust against Mutations and Noise. J. Theor. Biol. 253 (2), 323–332. 10.1016/j.jtbi.2008.03.003 18417154

[B35] Song H. Jiang Z. Men A. Yang B. (2017). A Hybrid Semi-supervised Anomaly Detection Model for High-Dimensional Data. Comput. Intelligence Neurosci. 2017, 1–9. 10.1155/2017/8501683 PMC5706085 29270197

[B36] Soon W. W. Hariharan M. Snyder M. P. (2013). High‐throughput Sequencing for Biology and Medicine. Mol. Syst. Biol. 9 (1), 640. 10.1038/msb.2012.61 23340846 PMC3564260

[B37] Suzuki Y. Lu M. Ben-Jacob E. Onuchic J. N. (2016). Periodic, Quasi-Periodic and Chaotic Dynamics in Simple Gene Elements with Time Delays. Sci. Rep. 6 (1), 21037. 10.1038/srep21037 26876008 PMC4753448

[B38] Tsimring L. S. (2014). Noise in Biology. Rep. Prog. Phys. 77 (2)–026601. 10.1088/0034-4885/77/2/026601 PMC4033672 24444693

[B39] Tyers M. Mann M. (2003). From Genomics to Proteomics. Nature 422 (6928), 193–197. 10.1038/nature01510 12634792

[B40] Wang H. Li M. Yue X. (2021). IncLSTM: Incremental Ensemble LSTM Model towards Time Series Data. Comput. Electr. Eng. 92, 107156. 10.1016/j.compeleceng.2021.107156

[B41] Wang W. Huang Y. Wang Y. Wang L. (2014). “Generalized Autoencoder: A Neural Network Framework for Dimensionality Reduction,” in Proceedings of the 2014 IEEE Conference on Computer Vision and Pattern Recognition Workshops, Columbus, OH, USA, June 2014. 10.1109/CVPRW.2014.79

[B42] Weckwerth W. (2003). Metabolomics in Systems Biology. Annu. Rev. Plant Biol. 54 (1), 669–689. 10.1146/annurev.arplant.54.031902.135014 14503007

[B43] Wu J. Vallenius T. Ovaska K. Westermarck J. Mäkelä T. P. Hautaniemi S. (2009). Integrated Network Analysis Platform for Protein-Protein Interactions. Nat. Methods 6 (1), 75–77. 10.1038/nmeth.1282 19079255

[B44] Wu W. Song L. Yang Y. Wang J. Liu H. Zhang L. (2020). Exploring the Dynamics and Interplay of Human Papillomavirus and Cervical Tumorigenesis by Integrating Biological Data into a Mathematical Model. BMC Bioinformatics 21 (7), 152. 10.1186/s12859-020-3454-5 32366259 PMC7199323

[B45] Xia Y. Yang C. Hu N. Yang Z. He X. Li T. (2017). Exploring the Key Genes and Signaling Transduction Pathways Related to the Survival Time of Glioblastoma Multiforme Patients by a Novel Survival Analysis Model. BMC Genomics 18 (1), 950. 10.1186/s12864-016-3256-3 28198665 PMC5310279

[B46] Xiao M. Liu G. Xie J. Dai Z. Wei Z. Ren Z. (2021). 2019nCoVAS: Developing the Web Service for Epidemic Transmission Prediction, Genome Analysis, and Psychological Stress Assessment for 2019-nCoV. Ieee/acm Trans. Comput. Biol. Bioinf. 18 (4), 1250–1261. 10.1109/TCBB.2021.3049617 PMC8769043 33406042

[B47] Xiao M. Yang X. Yu J. Zhang L. (2020). CGIDLA:Developing the Web Server for CpG Island Related Density and LAUPs (Lineage-Associated Underrepresented Permutations) Study. Ieee/acm Trans. Comput. Biol. Bioinf. 17 (6), 2148–2154. 10.1109/TCBB.2019.2935971 31443042

[B48] You Y. Ru X. Lei W. Li T. Xiao M. Zheng H. (2020). Developing the Novel Bioinformatics Algorithms to Systematically Investigate the Connections Among Survival Time, Key Genes and Proteins for Glioblastoma Multiforme. BMC Bioinformatics 21 (13), 383. 10.1186/s12859-020-03674-4 32938364 PMC7646399

[B49] Zhang A. Sun H. Wang P. Han Y. Wang X. (2012a). Recent and Potential Developments of Biofluid Analyses in Metabolomics. J. Proteomics 75 (4), 1079–1088. 10.1016/j.jprot.2011.10.027 22079244

[B50] Zhang Z. Ye W. Qian Y. Zheng Z. Huang X. Hu G. (2012b). Chaotic Motifs in Gene Regulatory Networks. PLOS ONE 7 (7), e39355. 10.1371/journal.pone.0039355 22792171 PMC3391214

[B51] Zhang L. Dai Z. Yu J. Xiao M. (2020). CpG-island-based Annotation and Analysis of Human Housekeeping Genes. Brief. Bioinform. 22 (1), 515–525. 10.1093/bib/bbz134 31982909

[B52] Zhang L. Fu C. Li J. Zhao Z. Hou Y. Zhou W. (2019a). Discovery of a Ruthenium Complex for the Theranosis of Glioma through Targeting the Mitochondrial DNA with Bioinformatic Methods. Ijms 20 (18), 4643. 10.3390/ijms20184643 PMC6770666 31546801

[B53] Zhang L. Li J. Yin K. Jiang Z. Li T. Hu R. (2019b). Computed Tomography Angiography-Based Analysis of High-Risk Intracerebral Haemorrhage Patients by Employing a Mathematical Model. BMC Bioinformatics 20 (7), 193. 10.1186/s12859-019-2741-5 31074379 PMC6509873

[B54] Zhang L. Liu G. Kong M. Li T. Wu D. Zhou X. (2019c). Revealing Dynamic Regulations and the Related Key Proteins of Myeloma-Initiating Cells by Integrating Experimental Data into a Systems Biological Model. Bioinformatics 37 (11), 1554–1561. 10.1093/bioinformatics/btz542 PMC8485849 31350562

[B55] Zhang L. Qiao M. Gao H. Hu B. Tan H. Zhou X. (2016). Investigation of Mechanism of Bone Regeneration in a Porous Biodegradable Calcium Phosphate (CaP) Scaffold by a Combination of a Multi-Scale Agent-Based Model and Experimental Optimization/validation. Nanoscale 8 (31), 14877–14887. 10.1039/C6NR01637E 27460959 PMC10150920

[B56] Zhang L. Xiao M. Zhou J. Yu J. (2018). Lineage-associated Underrepresented Permutations (LAUPs) of Mammalian Genomic Sequences Based on a Jellyfish-Based LAUPs Analysis Application (JBLA). Bioinformatics 34 (21), 3624–3630. 10.1093/bioinformatics/bty392 29762634

[B57] Zhang L. Zhang L. Guo Y. Xiao M. Feng L. Yang C. (2021a). MCDB: A Comprehensive Curated Mitotic Catastrophe Database for Retrieval, Protein Sequence Alignment, and Target Prediction. Acta Pharmaceutica Sinica B. 10.1016/j.apsb.2021.05.032 PMC8546929 34729303

[B58] Zhang L. Zhang S. (2017). Using Game Theory to Investigate the Epigenetic Control Mechanisms of Embryo Development. Phys. Life Rev. 20, 140–142. 10.1016/j.plrev.2017.01.007 28109753

[B59] Zhang L. Zhao J. Bi H. Yang X. Zhang Z. Su Y. (2021b). Bioinformatic Analysis of Chromatin Organization and Biased Expression of Duplicated Genes between Two Poplars with a Common Whole-Genome Duplication. Hortic. Res. 8 (1), 62. 10.1038/s41438-021-00494-2 33750794 PMC7943600

