# Supplementary material for: Developing an Embedding, Koopman and Autoencoder Technologies-Based Multi-Omics Time Series Predictive Model (EKATP) for Systems Biology research
Source: Front Genet. 2021 Oct 26;12:761629. doi: 10.3389/fgene.2021.761629 (PMC8576451; doi:10.3389/fgene.2021.761629)
Supplement: Supplementary file 1 [file Table1.docx]

# Supplementary Table 1

**Genomics dataset**

**Table 1.1** The data of low-dimensional gene expression time series $\left\{ v_{t} \right\}$ under the condition of $h$=0.003 and $T$=1050

The data is listed on <https://github.com/suranl/EKATP> (Supplementary Table 1.1.csv)

**Table 1.2** The data of low-dimensional gene expression time series $\left\{ v_{t} \right\}$ under the condition of $h$=0.006 and $T$=1050

The data is listed on <https://github.com/suranl/EKATP> (Supplementary Table 1.2.csv)

**Table 1.3** The data of low-dimensional gene expression time series with$T$=15000

The data is listed on <https://github.com/suranl/EKATP> (Supplementary Table 1.3.csv)

**Table 1.4** The data of high-dimensional gene expression time series $\left\{ f_{t} \right\}$ under the condition of $h$=0.003 and $T$=1050

The data is listed on <https://github.com/suranl/EKATP> (Supplementary Table 1.4.csv)

**Table 1.5** The data of high-dimensional gene expression time series $\left\{ f_{t} \right\}$ under the condition of $h$=0.006 and $T$=1050

The data is listed on <https://github.com/suranl/EKATP> (Supplementary Table 1.5.csv)

**Table 1.6** The data of high-dimensional gene expression time series with $T$=15000.

The data is listed on <https://github.com/suranl/EKATP> (Supplementary Table 1.6.csv)

**Table 1.7** Parameter setting of Lorenz system

| Parameter | $\eta$ | $\rho$ | $\beta$ |
| --- | --- | --- | --- |
| value | 10.0 | 28.0 | 8.0/3.0 |

**Table 1.8** Division of training set and testing set

| Datasets | Training set | Testing set |
| --- | --- | --- |
| Value | [0:1000] | [1000:1050] |

**Table 1.9** The interval of gene time series in three different periods

| Parameter | $T$ | $t_{1}$ | $t_{2}$ | $t_{3}$ |
| --- | --- | --- | --- | --- |
| value | 15000 | [2000:3000] | [2700:3700] | [5780:6580] |
