# Supplementary material for: Developing an Embedding, Koopman and Autoencoder Technologies-Based Multi-Omics Time Series Predictive Model (EKATP) for Systems Biology research
Source: Front Genet. 2021 Oct 26;12:761629. doi: 10.3389/fgene.2021.761629 (PMC8576451; doi:10.3389/fgene.2021.761629)
Supplement: Supplementary file 2 [file Presentation1.pdf]

Supplementary Presentation 1 Related works

1.1 Autoencoder

Autoencoder (Wang et al. 2014) is a class of neural network that can compress high-dimensional state into low-dimensional form by encoder χ_e and restore low-dimensional state to high-dimensional form by decoder χ_d.

Here, Eq. 1 shows how to map the high-dimensional inputting state $\boldsymbol{F^t}$ onto reconstructed high-dimensional state $\widehat{\boldsymbol{F}}^{\boldsymbol{t}}$,

$$\widehat{\boldsymbol{F}}^{\boldsymbol{t}} = \chi_d \circ \chi_e(\boldsymbol{F^t}) \tag{1}$$

where $\circ$ is the function composition operation.

Previously, Lusch et al. (Lusch, Kutz and Brunton 2018) build a deep auto-encoder framework to accurately predict the future state of metabolomics time series with flow behavior, which demonstrates that autoencoder is good at processing nonlinear systems.

1.2 Delay embedding theory

For a high dimensional nonlinear system with n-dimensional variables, we define $\boldsymbol{F^t} = (f_1^t, f_2^t, \dots, f_n^t)'$ as the observed non-delay attractor, which represents the state of the system at time step t in the n-dimensional space. Here "'" is the transpose of a vector. The delay embedding theory (Sauer, Yorke and Casdagli 1991, Holmes et al. 2012) suggests that, the mapping $\Phi: \mathbb{R}^n \to \mathbb{R}^L$ to the observed non-delay attractor $\boldsymbol{F^t}$ is an embedding when $L > 2d$ (d denotes the box-counting dimension of $\boldsymbol{F^t}$), and a delay attractor $\boldsymbol{Y^t} = (y^t, y^{t+1}, \dots, y^{t+L-1})'$ of length L can be constructed by Eq. 2.

$$\Phi(\boldsymbol{F^t}) = (y^t, y^{t+1}, \dots, y^{t+L-1})' = \boldsymbol{Y^t} \tag{2}$$

Moreover, the mapping between $\boldsymbol{F^t}$ and $\boldsymbol{Y^t}$ is a one-to-one map with the conjugate form, Chen et al. (Chen et al. 2020) has derived the conjugate form of Φ as $\Psi: \mathbb{R}^L \to \mathbb{R}^n$ (Eq. 3).

$$\Psi(\boldsymbol{Y^t}) = (f_1^t, f_2^t, \dots, f_n^t)' = \boldsymbol{F^t} \tag{3}$$

Previous studies have predicted the future state of multi-omics time series based on the delay embedding theory (Sauer et al. 1991, Holmes et al. 2012). For example, Chen et al. (Chen et al. 2020) proposed an Anticipated Learning Machine (ALM) to achieve precise future-state prediction of time series with chaotic behavior related to genomics.

1.3 Koopman theory

Koopman theory (Koopman 1931) provides a new research direction in terms of dealing with complex nonlinear relations, which suggests that a nonlinear dynamical system can undergo a transformation into an infinite dimensional space, in which it evolves linearly in time by virtue of Koopman operator (Mezić 2005, Budišić, Mohr and Mezić 2012).

However, we need to find an approximate finite dimensional representation for the infinite dimensional Koopman operator in practice. Rice et al. (Rice, Xu and August 2020) assumed that there exists a mapping that can approximate Koopman operator to a finite dimension linear matrix, which can learn the forward (or backward) dynamics of system. Based on Koopman theory, Azencot et al. (Azencot et al. 2020) develop a Physics Constrained Learning framework to accurately predict the future state of proteomics time series data with oscillating behavior metabolomics.

Reference:

Azencot, O., N. B. Erichson, V. Lin and M. Mahoney (2020). Forecasting sequential data using consistent Koopman autoencoders. International Conference on Machine Learning.

Budišić, M., R. Mohr and I. Mezić (2012). "Applied koopmanism." Chaos: An Interdisciplinary Journal of Nonlinear Science **22**(4): 047510.

Chen, C., R. Li, L. Shu, Z. He, J. Wang, C. Zhang, H. Ma, K. Aihara and L. Chen (2020). "Predicting future dynamics from short-term time series using an Anticipated Learning Machine." National Science Review **7**(6): 1079-1091.

Chen, P., R. Liu, K. Aihara and L. Chen (2020). "Autoreservoir computing for multistep ahead prediction based on the spatiotemporal information transformation." Nature communications **11**(1): 1-15.

Holmes, P., J. L. Lumley, G. Berkooz and C. W. Rowley (2012). Turbulence, coherent structures, dynamical systems and symmetry, Cambridge university press.

Koopman, B. O. (1931). "Hamiltonian systems and transformation in Hilbert space." Proceedings of the national academy of sciences of the united states of america **17**(5): 315.

Lusch, B., J. N. Kutz and S. L. Brunton (2018). "Deep learning for universal linear embeddings of nonlinear dynamics." Nature communications **9**(1): 1-10.

Mezić, I. (2005). "Spectral properties of dynamical systems, model reduction and decompositions." Nonlinear Dynamics **41**(1): 309-325.

Rice, J., W. Xu and A. August (2020). "Analyzing Koopman approaches to physics-informed machine learning for long-term sea-surface temperature forecasting." arXiv preprint arXiv:2010.00399.

Sauer, T., J. A. Yorke and M. Casdagli (1991). "Embedology." Journal of statistical Physics **65**(3): 579-616.

Wang, W., Y. Huang, Y. Wang and L. Wang (2014). Generalized autoencoder: A neural network framework for dimensionality reduction. Proceedings of the IEEE conference on computer vision and pattern recognition workshops.
