# Supplementary material for: Developing an Embedding, Koopman and Autoencoder Technologies-Based Multi-Omics Time Series Predictive Model (EKATP) for Systems Biology research
Source: Front Genet. 2021 Oct 26;12:761629. doi: 10.3389/fgene.2021.761629 (PMC8576451; doi:10.3389/fgene.2021.761629)
Supplement: Supplementary file 3 [file Presentation4.pdf]

Supplementary Presentation 4 Evaluation method

To evaluate the experimental results, we use the relative predictive error, which is computed at each time step t via Eq. 1:

$$Predictive\ error = \left\|\boldsymbol{F_t} - \widehat{\boldsymbol{F}}_{\boldsymbol{t}}\right\|_2 / \|\boldsymbol{F_t}\|_2 \tag{1}$$

where $\boldsymbol{F_t}$ represents true state, $\widehat{\boldsymbol{F}}_{\boldsymbol{t}}$ represents predictive state. In EKATP model and KAE model (Azencot et al. 2020), we average the errors over 30 different initial observations, where the shaded areas represent the ± 1 standard deviations.

P.S. Our code is available at https://github.com/suranl/EKATP

Reference:

Azencot, O., N. B. Erichson, V. Lin and M. Mahoney (2020). Forecasting sequential data using consistent Koopman autoencoders. International Conference on Machine Learning.
