# Supplementary material for: Developing an Embedding, Koopman and Autoencoder Technologies-Based Multi-Omics Time Series Predictive Model (EKATP) for Systems Biology research
Source: Front Genet. 2021 Oct 26;12:761629. doi: 10.3389/fgene.2021.761629 (PMC8576451; doi:10.3389/fgene.2021.761629)
Supplement: Supplementary file 4 [file Table2.docx]

# Supplementary Table 2

**Proteomics dataset**

**Table 2.1** The data of low-dimensional protein time series $\left\{ v_{t} \right\}$ under the condition of $h$=0.8 and $T$=1600

The data is listed on <https://github.com/suranl/EKATP> (Supplementary Table 2.1.csv)

**Table 2.2** The data of low-dimensional protein time series $\left\{ v_{t} \right\}$ under the condition of $h$=2.4 and $T$=1600

The data is listed on <https://github.com/suranl/EKATP> (Supplementary Table 2.2.csv)

**Table 2.3** The data of high-dimensional protein time series $\left\{ v_{t} \right\}$ under the condition of $h$=0.8 and $T$=1600

The data is listed on <https://github.com/suranl/EKATP> (Supplementary Table 2.3.csv)

**Table 2.4** The data of high-dimensional protein time series $\left\{ v_{t} \right\}$ under the condition of $h$=2.4 and $T$=1600

The data is listed on <https://github.com/suranl/EKATP> (Supplementary Table 2.4.csv)

**Table 2.5** Parameter setting of pendulum system

| Parameter | $l$ | $g$ |
| --- | --- | --- |
| value | 1 | 9.8 |

**Table 2.6** Division of training set and testing set

| Datasets | Training set | Testing set |
| --- | --- | --- |
| Value | [0:600] | [600:1600] |

**Table 2.7** The seed value of each condition

| Condition | $h$=0.8  $\sigma$=0.00 | $h$=0.8  $\sigma$=0.03 | $h$=2.4  $\sigma$=0.00 | $h$=2.4  $\sigma$=0.00 |
| --- | --- | --- | --- | --- |
| Seed value | 1-20 | 21-40 | 121-140 | 141-160 |
