# Supplementary material for: Developing an Embedding, Koopman and Autoencoder Technologies-Based Multi-Omics Time Series Predictive Model (EKATP) for Systems Biology research
Source: Front Genet. 2021 Oct 26;12:761629. doi: 10.3389/fgene.2021.761629 (PMC8576451; doi:10.3389/fgene.2021.761629)
Supplement: Supplementary file 5 [file Table3.docx]

**Supplementary Table 3**

**Metabolomics dataset**

**Table 3.1** The data of low-dimensional metabolic time series $\left\{ v_{t} \right\}$ under the condition of $\zeta_{1}$ and $T$=900 (Lusch et al. 2018)

The data is listed on <https://github.com/suranl/EKATP> (Supplementary Table 3.1.csv)

**Table 3.2** The data of low-dimensional metabolic time series $\left\{ v_{t} \right\}$ under the condition of $\zeta_{2}$ and $T$=900 (Lusch et al. 2018)

The data is listed on <https://github.com/suranl/EKATP> (Supplementary Table 3.2.csv)

**Table 3.3** The data of high-dimensional metabolic time series $\left\{ v_{t} \right\}$ under the condition of $\zeta_{1}$ and $T$=900

The data is listed on <https://github.com/suranl/EKATP> (Supplementary Table 3.3.csv)

**Table 3.4** The data of high-dimensional metabolic time series $\left\{ v_{t} \right\}$ under the condition of $\zeta_{2}$ and $T$=900

The data is listed on <https://github.com/suranl/EKATP> (Supplementary Table 3.4.csv)

**Table 3.5** Parameter setting of fluid flow system

| Parameter | $\gamma$ | $\omega$ | $A$ | $\lambda$ |
| --- | --- | --- | --- | --- |
| Value | 0.1 | 1 | -0.1 | 10 |

**Table 3.6** Division of training set and testing set

| Dataset | Training set | Testing set |
| --- | --- | --- |
| Value | [0:800] | [800:900] |

**Table 3.7** PCC and RMSE values between predictive state and true state under different noise intensities

| $\sigma$ | 0.001 | 0.005 | 0.010 | 0.050 | 0.100 | 0.500 |
| --- | --- | --- | --- | --- | --- | --- |
| PCC | 0.999818 | 0.999726 | 0.999418 | 0.945674 | 0.768908 | 0.166338 |
| RMSE | 0.000225 | 0.000254 | 0.000332 | 0.003008 | 0.006405 | 0.015410 |
