# Supplementary material for: Developing an Embedding, Koopman and Autoencoder Technologies-Based Multi-Omics Time Series Predictive Model (EKATP) for Systems Biology research
Source: Front Genet. 2021 Oct 26;12:761629. doi: 10.3389/fgene.2021.761629 (PMC8576451; doi:10.3389/fgene.2021.761629)
Supplement: Supplementary file 6 [file Presentation2.pdf]

Supplementary Presentation 2   Experimental setup

We set the experimental environment as follows: we use deep learning framework PyTorch to implement the EKATP. The experimental hardware environment is configured by Intel(R) Core (TM) i5-10210U CPU @ 1.60GHz, the memory of which is 16.0 GB.

Our experiment employs the following benchmark methods for predictive performance comparison, which are recurrent neural network (RNN) (Jiang and Lai 2019), long short-term memory (LSTM) (Hochreiter and Schmidhuber 1997), dynamic Autoencoder (DAE) (Lusch, Kutz and Brunton 2018) and Koopman Autoencoder (KAE) (Azencot et al. 2020). We detail these methods as follows.

RNN (Jiang et al. 2019) is a classical deep neural network, usually used for sequential learning tasks; LSTM (Hochreiter et al. 1997) is the variant of the classical RNN, which introduces the concept of cell state to improve the structure of RNN; DAE (Lusch et al. 2018) develops the deep neural network representations of Koopman eigenfunctions, which is often used for high-dimensional and nonlinear systems; KAE (Azencot et al. 2020) is a physically constrained learning model based on the Koopman theory for high-dimensional time series data processing.

Reference:

Azencot, O., N. B. Erichson, V. Lin and M. Mahoney (2020). Forecasting sequential data using consistent Koopman autoencoders. International Conference on Machine Learning.

Hochreiter, S. and J. Schmidhuber (1997). "Long short-term memory." Neural computation **9**(8): 1735-1780.

Jiang, J. and Y.-C. Lai (2019). "Model-free prediction of spatiotemporal dynamical systems with recurrent neural networks: Role of network spectral radius." Physical Review Research **1**(3): 033056.DOI: 10.1103/PhysRevResearch.1.033056.

Lusch, B., J. N. Kutz and S. L. Brunton (2018). "Deep learning for universal linear embeddings of nonlinear dynamics." Nature communications **9**(1): 1-10.
