# Supplementary material for: Developing an Embedding, Koopman and Autoencoder Technologies-Based Multi-Omics Time Series Predictive Model (EKATP) for Systems Biology research
Source: Front Genet. 2021 Oct 26;12:761629. doi: 10.3389/fgene.2021.761629 (PMC8576451; doi:10.3389/fgene.2021.761629)
Supplement: Supplementary file 7 [file Presentation3.pdf]

Supplementary Presentation 3 Pseudocode of the EKATP

3.1 Pseudocode of training process

1:	**Input:** High-dimensional nonlinear time series state $\boldsymbol{F^t} = (f_1^t, f_2^t, \dots f_n^t)'$ of training set, number of epochs: e, predictive step: k	
2	**For** $e^0 = 0, \dots, e$	
3:	$\boldsymbol{Y^t} \leftarrow \chi_e(\boldsymbol{F^t})$	obtain low-dimensional state at time t by (2)
	$\boldsymbol{Y}^{t+s} \leftarrow C^s \boldsymbol{Y^t}, s \in [1, k]$	obtain low-dimensional state at time $t+s$ by (8)
	$\boldsymbol{Y}^{t-s} \leftarrow D^s \boldsymbol{Y^t}, s \in [1, k]$	obtain low-dimensional state at time $t-s$ by (9)
	$\boldsymbol{\hat{F}^{t\pm s}} \leftarrow \chi_d(\boldsymbol{Y^{t\pm s}}), s \in [1, k]$	obtain high-dimensional state at time $t \pm s$ by (10)
	Calculate the loss function by (15)	
	**End For**	
4:	**Output:** trained EKATP	

3.2 Pseudocode of testing process

1:	**Input:** High-dimensional nonlinear time series state $\boldsymbol{F^t} = (f_1^t, f_2^t, \dots f_n^t)'$ of testing set, predictive step: p, trained EKATP	
2:	$\boldsymbol{Y^t} \leftarrow \chi_e(\boldsymbol{F^t})$	obtain low-dimensional state at time t by (2)
	$\boldsymbol{Y}^{t+s} \leftarrow C^s \boldsymbol{Y^t}, s \in [1, p]$	obtain low-dimensional state at time $t+s$ by (8)
	$\boldsymbol{\hat{F}^{t+s}} \leftarrow \chi_d(\boldsymbol{Y^{t+s}}), s \in [1, p]$	obtain high-dimensional state at time $t+s$ by (10)
3:	**Output:** High-dimensional predictive state $\boldsymbol{\hat{F}^{t+s}}, s \in [1, p]$	
