# Supplementary figures and images for: Developing an Embedding, Koopman and Autoencoder Technologies-Based Multi-Omics Time Series Predictive Model (EKATP) for Systems Biology research
Source: Front Genet. 2021 Oct 26;12:761629. doi: 10.3389/fgene.2021.761629 (PMC8576451; doi:10.3389/fgene.2021.761629)

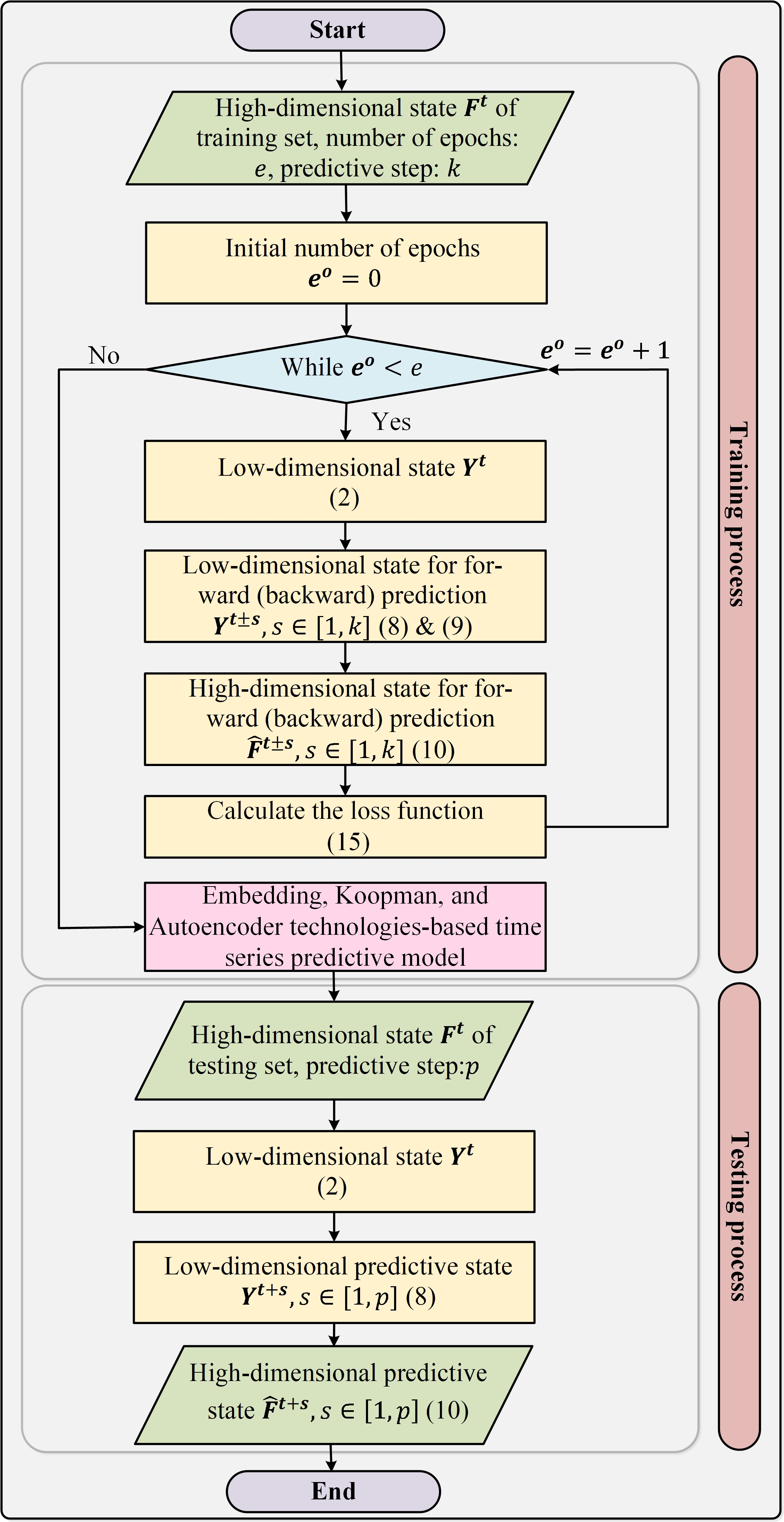

Supplement: Supplementary file 8 [file Image1.jpg]
